# Serum cystatin C as a potential biomarker for generalized acetylcholine receptor antibody-positive myasthenia gravis

**DOI:** 10.3389/fimmu.2025.1578359

**Published:** 2025-05-08

**Authors:** Dingxian He, Huahua Zhong, Lei Jin, Ran Chen, Zongtai Wu, Rui Zhao, Xiao Huan, Chong Yan, Jie Song, Jianying Xi, Chongbo Zhao, Shanfeng Zhu, Sushan Luo

**Affiliations:** ^1^ Huashan Rare Disease Centre and Department of Neurology, Huashan Hospital, Shanghai Medical College, National Centre for Neurological Disorders, Fudan University, Shanghai, China; ^2^ Department of Public Health and Primary Care, University of Cambridge, Cambridge, United Kingdom; ^3^ Institute of Science and Technology for Brain-Inspired Intelligence and Ministry of Education (MOE) Frontiers Center for Brain Science, Fudan University, Shanghai, China; ^4^ Key Laboratory of Computational Neuroscience and Brain-Inspired Intelligence (Fudan University), Ministry of Education, Shanghai, China; ^5^ Zhangjiang Fudan International Innovation Center, Fudan University, Shanghai, China

**Keywords:** myasthenia gravis, cystatin C, biomarker, T helper cell, clinical severity

## Abstract

**Objective:**

To identify new metabolic biomarkers associated with myasthenia gravis (MG).

**Methods:**

We analyzed 285 potential metabolic molecules from UK Biobank (UKB) for MG patients and identified elevated serum cystatin C (Cys-C). Validation was performed using laboratory data, ELISA, and clinical information from Chinese (CHN) acetylcholine receptor antibody (AChR-Ab) positive generalized MG (gMG) cohorts. We assessed cytokines/chemokines/complements and peripheral blood T lymphocytes using Luminex assays and flow cytometry. MG-relevant scores including myasthenia gravis activities of daily living score (MG-ADL) and quantitative myasthenia gravis score (QMG) were prospectively collected and retrospectively analyzed. The correlations between serum Cys-C and the ratio of T helper 1 (Th1)/Th2 were assessed.

**Results:**

Serum Cys-C levels were significantly elevated in MG patients compared to healthy controls in both UKB cohorts and Chinese MG cohorts (CHN) (UKB: 0.99 ± 0.20 vs. 0.86 ± 0.12 mg/L, p = 2.26E-41; CHN: 1.08 ± 0.30 vs. 0.87 ± 0.13 mg/L, p = 4.83E-08). Higher serum Cys-C levels were found in MG patients with high disease burden, as stratified by MG-ADL score. Serum Cys-C correlated with MG scores, including QMG (R = 0.40, p = 3.90E-03) and MG-ADL scores (R = 0.42, p = 2.40E-03). The ratio of Th1/Th2 correlated well with the serum Cys-C (R = 0.29, p = 3.10E-02).

**Conclusions:**

Serum Cys-C levels were significantly elevated in AChR-Ab positive gMG patients and correlated with disease severity and Th1/Th2 ratio, suggesting its potential as an efficient biomarker for predicting the clinical severity of MG. Future prospective cohort studies with a large sample size are expected to validate these findings.

## 1 Introduction

Myasthenia gravis (MG) is a T-cell-dependent, B-cell-mediated autoimmune disease caused by antibodies targeting the nicotinic acetylcholine receptor (AChR) or other components of the post-synaptic muscle endplate at the neuromuscular junction (NMJ) (1). The clinical presentation of MG ranges from an isolated ocular form to severe weakness of the limb, bulbar and respiratory muscles. The age of onset varies from childhood to late adulthood, with disease peaks observed in younger adult women and older men (2). MG represents a growing burden globally due to its increasing prevalence, substantial healthcare costs, reduced quality of life, and the significant psychosocial challenges it poses on patients and caregivers (3–5).

From both clinical and immunological perspectives, MG is a heterogeneous autoimmune disorder. The identification of reliable biomarkers is promising for improving diagnostic accuracy, distinguishing MG from other diseases associated with muscle weakness, guiding therapeutic decision-making, and evaluating prognosis (2). Previous studies have shown that delayed treatment or poor adherence can worsen the disease burden in MG, with around 60% of patients initially presenting with ocular symptoms eventually progressing to generalized MG. Kimiaki et al. introduced the concept of early fast therapy, highlighting that repeated strategies such as plasma exchange or intravenous immunoglobulin enabled MG patients to more frequently and rapidly achieve minimal manifestations or better, while maintaining prednisolone at ≤5 mg/day for ≥6 months (6). Autoantibodies against the components at the NMJ serve as sensitive, specific, and well-established diagnostic markers for MG. Approximately 80% of MG patients present with autoantibodies against AChR, while a smaller subset is seropositive for antibodies targeting muscle-specific kinase (MuSK), low-density lipoprotein receptor-related protein 4 (LRP4), or agrin (2). Additionally, AChR-antibody (AChR-Ab) titers have also been used as a biomarker for the therapeutic response of immunotherapies (7), although the absolute levels of these autoantibodies do not correlate with disease severity (8).

Beyond pathogenic autoantibodies, various potential biomarkers have been identified to be associated with MG, including microRNAs (e.g., miR-146), inflammatory cytokines (e.g., IFN-γ, IL-21), complement segments (e.g., C5a), and serum metabolites (e.g., arachidonic acid) (9–12). However, these findings were restrained to insufficient validation or small sample sizes. The emergence of high-throughput omics profiling techniques has facilitated biomarker discovery, with genome-wide association studies (GWAS) identifying specific HLA variants (e.g., HLA-B*08:01) and proteomic analyses revealing ITIH3 as a potential biomarker (13, 14). Peripheral metabolomics, offering an accessible approach to evaluate inflammatory status in MG patients, represents a promising yet unexplored avenue for biomarker discovery.

**Figure 1:**
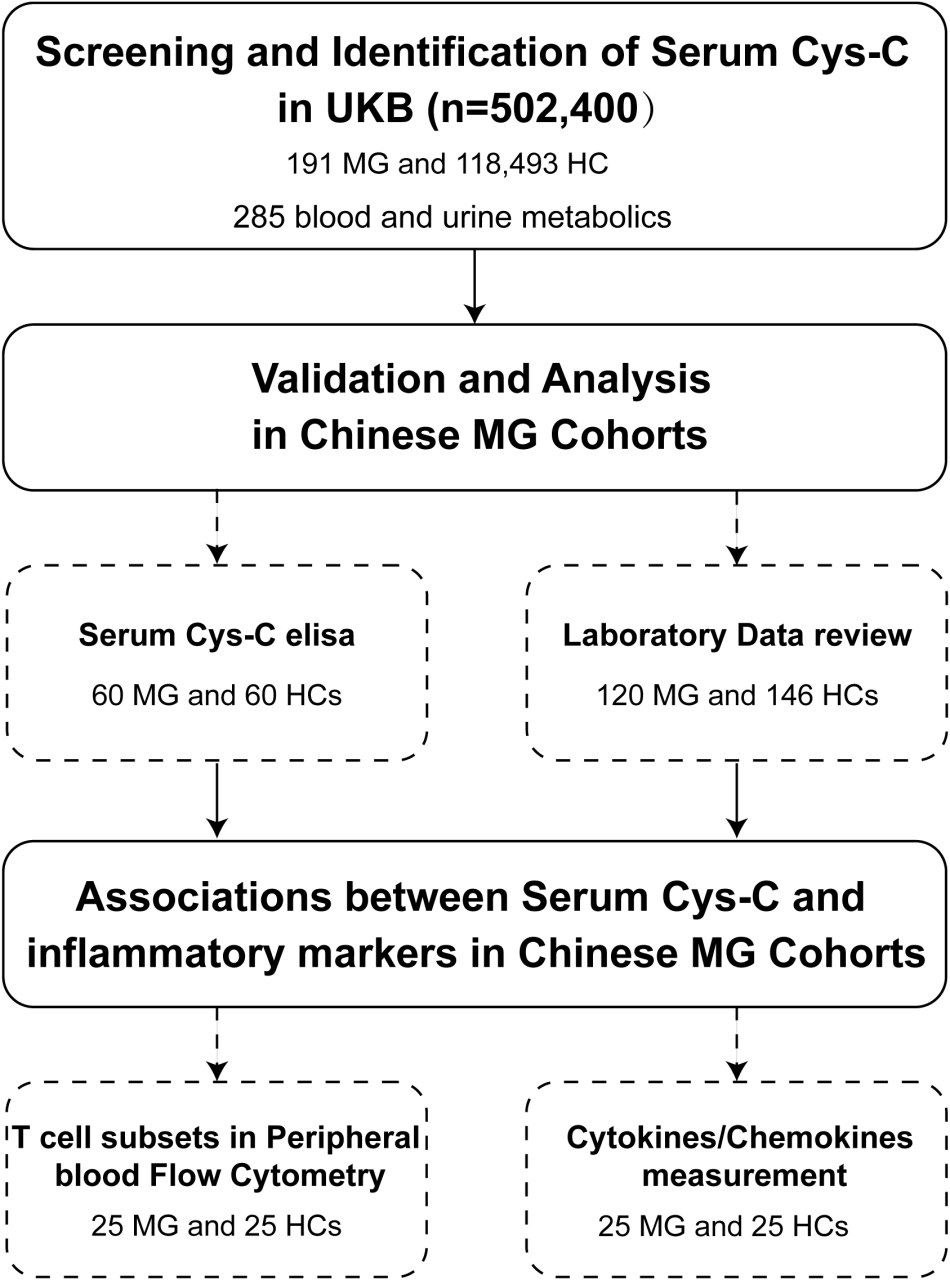
Flowchart of patient enrollment in this study. This flowchart illustrates the process of screening and identification of serum Cys-C as a potential biomarker for MG in the UKB cohort (n = 502,400), including 191 MG patients and 118,493 HCs, with a total of 285 metabolic molecules analyzed. Validation and further analyses were conducted in Chinese MG cohorts, involving serum Cys-C measurements (60 MG patients and 60 HCs) and laboratory data review (120 MG patients and 146 HCs). Additionally, associations between serum Cys-C levels and inflammatory markers were investigated, including peripheral blood T-cell subset flow cytometry and cytokine/chemokine measurements in a subset of 25 MG patients and 25 HCs. Cys-C, Cystatin C; UKB, UK Biobank; MG, myasthenia gravis; HC, health control.

In this study, we leveraged metabolic screening data from the UK Biobank (UKB) cohort of 502,400 adults and an independent Chinese MG cohort of 120 patients to: (1) identify novel peripheral biomarkers associated with the MG; (2) validate these biomarkers in an independent MG cohort and assess the associations with clinical and immunological parameters (**Figure 1**).

## 2 Methods

### 2.1 Screening for potential metabolic biomarkers for MG in UKB

UKB is a large-scale, population-based prospective cohort study designed to investigate the genetic, environmental, and lifestyle determinants of a wide range of diseases and health outcomes (15), which has been relatively underutilized in research on MG to date. We retrieved 285 potential metabolic molecules that have been screened in patients with MG from UKB, including 30 from blood biochemistry, 251 from nuclear magnetic resonance (NMR) metabolomics, and four from urine assays (**Supplementary Table 1**). MG patients were identified using UKB codes 1260 and 1437 based on self-reported illness data (https://biobank.ndph.ox.ac.uk/ukb/field.cgi?id=20002). Descriptions provided by patients were verified by a clinician and, where possible, cross-referenced with entries in the coding tree. Only the baseline group (Instance 0) was used for analysis, as given the limited representation of MG cases in later instances. We categorized all participants (n = 502,409) by their illness code into three groups: (1) MG (n = 191), (2) Healthy control (HC, n = 118,493), and (3) Other diseases (defined as non-MG, n = 383,716). Subsequently, a primary screening was conducted by comparing the p-values of MG vs. HC, followed by a secondary comparison of MG vs. non-MG controls. The UKB received ethical approval from the North West Multicenter Research Ethics Committee, and all participants provided written informed consent. This study was performed under the UKB application number 19542.

### 2.2 Validation for the identified biomarker and cytokine measurement in MG

From July to December 2024, 60 patients with anti-AChR-Ab generalized MG (gMG) were enrolled from the prospective registered cohort (NCT04535843), alongside 60 age- and sex-matched HCs. The study protocol was approved by the Ethics Committees of Huashan Hospital (2019–441 and 2022-913), and participants provided written informed consent. The basic demographic information and clinical features including sex, age at screening, onset age, thymoma status, thymectomy history, the clinical subtypes, myasthenia gravis activities of daily living score (MG-ADL), quantitative myasthenia gravis score (QMG), myasthenia gravis composite score (MGC), MG quality of life measure score (MG-QOL-15r) were collected (16–18). Peripheral blood serum samples were obtained from both MG patients and HCs and stored at -80°C refrigerator before analysis. MG patients were stratified into high-burden (MG-ADL score ≥6) and low-burden (MG-ADL <6) groups based on MG-ADL scores at enrollment (19).

Serum Cys-C levels were measured using an ELISA kit (Beyotime, China; PC220 Human Cys-C ELISA Kit). Twenty-two cytokines, chemokines, and complements were selected based on a literature review, including TNF-α, IFN-α, IFN-β, IFN-γ, IL-4, IL-6, IL-18, IL-17, IL-23, IL-2, IL-10, IL-19, IL-8, CCL3, CXCL2, CXCL4, CXCL5, BAFF and APRIL, C2, C5a and C9 (20–22). These were analyzed using two independent Human Luminex Discovery Assay panels (R&D Systems). Plasma samples were transferred into wells containing immobilized antibodies that capture the target molecules of interest. The Luminex assay results were analyzed on a Luminex^®^ 100/200™ instrument (Luminex Corporation). Cytokine measurements were obtained using a single replicate per sample, with values below the detection threshold recorded as 0 pg/ml. If more than 30% of cytokine results for a sample reported low quality, a backup plasma sample was retrieved and repeatedly tested.

### 2.3 Retrospective analysis of laboratory data in a Chinese MG cohort

We then retrospectively reviewed the laboratory biochemical data and the peripheral mononuclear cell flow cytometry from a Chinese cohort of gMG patients (n=120) and HCs (n=146) from the Department of Neurology, Huashan Hospital in 2024. HCs had no history of MG or other chronic inflammatory/autoimmune diseases. To eliminate the potential confounding effects of immunosuppressants on renal function, we enrolled the patients with treatment-naive gMG, as they had not received any immunotherapies at the time of assessment (n=35).

Laboratory data collected included: (1) renal function indicators in blood metabolic panel such as serum Cys-C, estimated glomerular filtration rate (eGFR, calculated by CKD-EPI creatinine equation), blood urea nitrogen (BUN), and uric acid (UA) (23); and (2) peripheral blood T lymphocyte flow cytometry results, specifically the absolute counts of Th1 and Th2 cells and their relative proportions among Th cells. The Th cell subsets were identified as follows: Th: CD3+CD4+, Th1: CXCR3-CCR6-; Th2: CXCR3-CCR6+. All laboratory analyses were performed and reported by the same centralized laboratory at Huashan Hospital.

### 2.4 Statistical analysis

Continuous variables were reported as mean ± standard deviation (SD) and compared using T-tests or analysis of variance (ANOVA). Categorical variables were reported as percentages (%) and compared using the chi-square test or Fisher’s exact test, depending on sample size. Correlation analysis was conducted using the Pearson method. In cases where multiple comparisons were made, the False Discovery Rate (FDR) method was applied to adjust p-values. The Wilcoxon signed-rank test was performed to compare differences between groups.

## 3 Results

### 3.1 Identification of elevated serum Cys-C in MG

**Figure 2:**
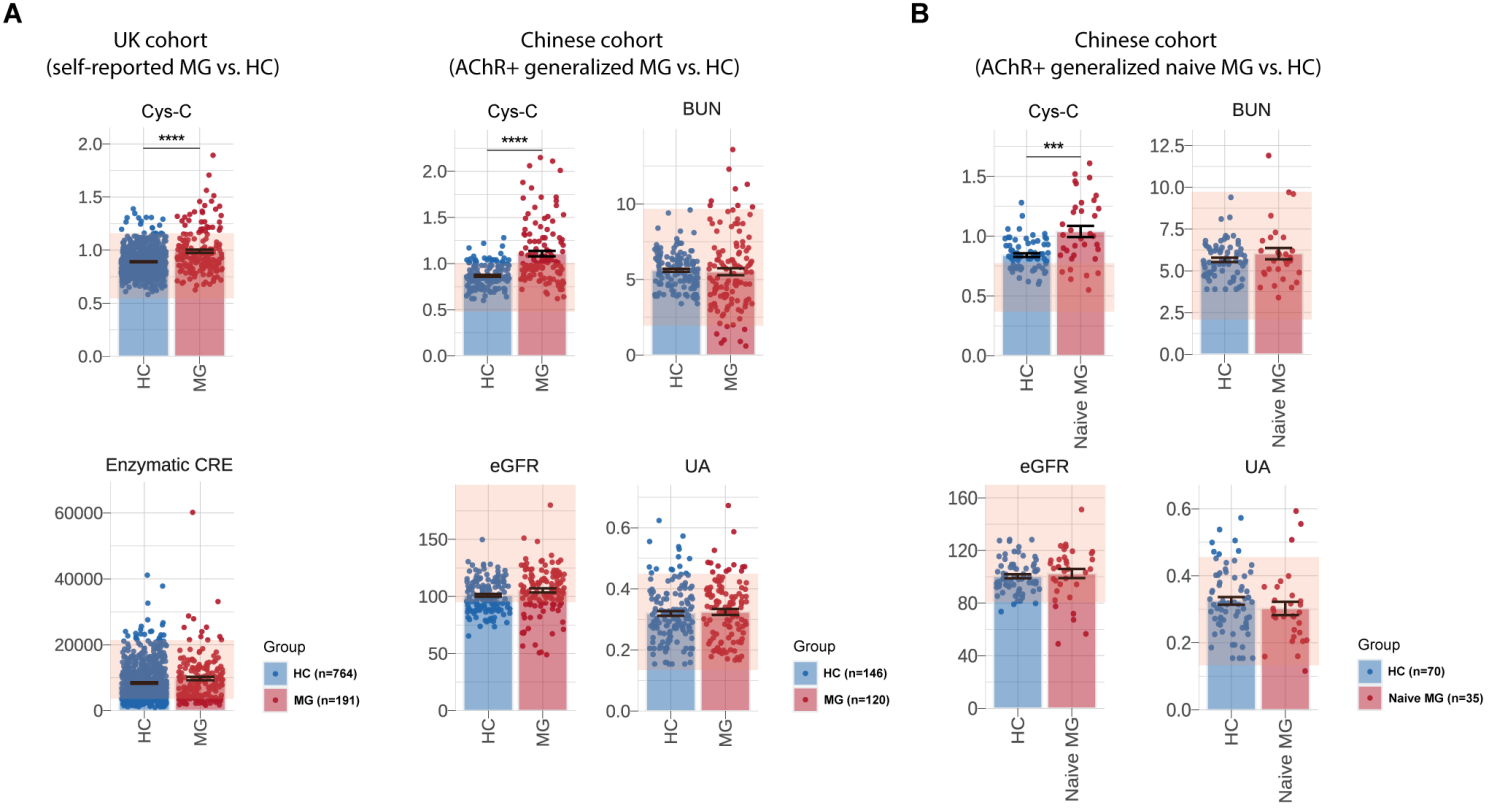
Measurement of serum Cys-C levels and renal function-related indicators of UK and Chinese cohort. Comparison of serum Cys-C and renal function-related indicators levels, including eGFR, UA, BUN and enzymatic CRE, between MG patients and HCs in the UK cohort (self-reported MG: N = 191, HCs: N = 118,493) and the CHN AChR-Ab+ gMG cohort (MG: N = 120, HCs: N = 146). **(B)** Comparison of serum Cys-C and renal function-related indicators levels, including eGFR, UA and BUN, between naive CHN gMG patients (N=35) and HCs (N=70). MG, myasthenia gravis; HC, health control; eGFR, estimated glomerular filtration rate; BUN, blood urea nitrogen; UA, uric acid; CRE, creatinine; Cys-C, Cystatin C; CHN, chinese; gMG, generalize myasthenia gravis. “***” indicates p < 0.001, and “****” indicates p < 0.0001.

In this study, by leveraging the metabolic screening data from the large UKB cohort (n = 502,400), we classified participants into three groups: MG patients (n = 191), healthy controls (HC, n = 118,493), and individuals with other diseases (n = 383,716) (**Table 1**). Serum Cystatin C (Cys-C) levels were significantly higher in MG patients compared to HCs (MG vs. HCs, 0.99 ± 0.20 vs. 0.86 ± 0.12 mg/L, p = 2.26E-41, adjusted p = 1.18E-40), ranking as the most prominently elevated biomarker among all 363 molecules analyzed. Furthermore, MG patients exhibited markedly higher serum Cys-C levels compared to patients with other diseases (MG vs. Other diseases, 0.99 ± 0.20 vs. 0.92 ± 0.19 mg/L, p = 2.88E-06, adjusted p = 1.02E-05), suggesting that serum Cys-C represents a distinctive metabolic signature in MG patients (**Figure 2A**).

**Table 1:** The top ten elevated molecules identified in MG from the UKB.

Candidate	MG (n=191)		HC (n=118493)		Other Diseases^1^ (n=383716)		p value			Adjusted p value		
	Mean	SD	Mean	SD	Mean	SD	p value (MG vs. HC)	p value (MG vs. Other Diseases)	p value (HC vs. Other Diseases)	Adjusted p value (MG vs. HC)	Adjusted p value (MG vs. Other Diseases)	Adjusted p value (HC vs. Other Diseases)
Cys_C	0.99	0.20	0.86	0.12	0.92	0.19	2.26E-41	2.88E-06	0	1.18E-40	1.02E-05	0
Glu	5.44	1.76	4.93	0.79	5.18	1.35	1.51E-17	1.19E-02	0	6.33E-17	2.84E-02	0
HbA1c	37.14	6.83	34.53	4.47	36.62	7.27	2.43E-15	3.28E-01	0	9.83E-15	4.54E-01	0
TP	70.47	4.32	72.50	3.99	72.52	4.15	3.31E-11	1.17E-10	2.27E-01	1.30E-10	4.54E-10	3.45E-01
ALB	44.30	2.56	45.44	2.53	45.14	2.66	4.30E-09	3.61E-05	2.99E-215	1.62E-08	1.19E-04	4.23E-214
AST	29.18	40.62	25.27	9.25	26.52	11.00	1.94E-08	1.15E-03	2.54E-254	7.19E-08	3.24E-03	5.07E-253
NMRm_23605^2^	50.13	4.82	52.78	3.80	51.11	4.73	1.91E-06	1.58E-01	0	6.77E-06	2.58E-01	0
NMRm_23608^3^	20.46	4.31	18.16	3.37	19.62	4.20	2.86E-06	1.70E-01	0	1.01E-05	2.75E-01	0
NMRm_23606^4^	34.24	4.38	36.58	3.45	35.04	4.26	3.55E-06	1.99E-01	0	1.25E-05	3.12E-01	0
NMRm_23613^5^	9.01	2.50	7.72	1.95	8.59	2.56	5.86E-06	2.60E-01	4.70E-228	2.10E-05	5.40E-02	7.79E-227

^1^Diseases except for myasthenia gravis.

^2^Cholesterol to total lipids in very small VLDL percentage.

^3^Triglycerides to total lipids in very small VLDL percentage.

^4^Cholesteryl esters to total lipids in very small VLDL percentage.

^5^Triglycerides to total lipids in IDL percentage.

Cys-C, Cystatin C; Glu, blood glucose; HbA1c, glycated hemoglobin A1c; TP, total protein; ALB, albumin; AST, aspartate aminotransferase; MG, myasthenia gravis; HC, health control; NMR, nuclear magnetic resonance; VLDL, very low-density lipoprotein; IDL, intermediate-density lipoprotein.

### 3.2 Validation of serum Cys-C levels in an independent Chinese MG cohort

In an independent Chinese (CHN) gMG cohort, 120 AChR-Ab positive gMG patients and 146 HCs were enrolled in this study (**Table 2**). The sex (CHN gMG vs. HCs, men%/women%, 30.12%/69.88% vs. 43.15%/56.85%, p =6.60E-02) and age (CHN gMG vs. HCs, 53.08 ± 18.89 vs. 48.13 ± 13.40 years, p =6.05E-02) distributions were comparable between the two groups. Compared with HCs, CHN gMG patients exhibited elevated serum Cys-C levels (CHN gMG vs. HCs, 1.08 ± 0.30 vs. 0.87 ± 0.13mg/L, p = 4.83E-08). Since Cys-C is mainly involved or associated with renal glomerular filtration, we next analyzed the impact of renal impairment in patients with MG by utilizing CHN gMG cohort and MG patients from UKB (24). The renal function-related indicators, including eGFR (CHN gMG vs. HCs, 105.30 ± 20.66 vs. 101.01 ± 14.33ml/min, p = 5.17E-02), Uric Acid (UA) (CHN gMG vs. HCs, 0.27 ± 0.07 vs. 0.32 ± 0.09mmol/L, p = 6.85E-01), BUN (CHN gMG vs. HCs, 6.64 ± 2.41 vs. 5.61 ± 1.17mmol/L, p = 7.17E-01) and enzymatic CRE (UKB MG vs. UKB HCs, 9760.00 ± 7047.00 vs. 8394.00 ± 5658.00μmol/L, p = 5.70E-02), showed no significant differences between MG and HCs in both CHN gMG and UKB MG cohort (**Figure 2A**).

**Table 2:** Clinical characteristics and renal function-related indicators of UK and Chinese cohort.

Feature	UK cohort			Chinese cohort		
	HC (n=764)	MG^1^ (n=191)	p value	HC (n=146)	MG (n=120)	p value
Sex, % men/% women	42.93%/57.07%	42.93%/57.07%	1.00E+00	43.15%/56.85%	30.12%/69.88%	6.60E-02
Age (years), mean ± SD	59.49 ± 6.79	59.49 ± 6.80	1.00E+00	48.13 ± 13.40	53.08 ± 18.89	6.05E-02
AChR positive, n (%)	/	/	/	/	120 (100%)	/
Cys-C (mg/L), mean ± SD	0.89 ± 0.12	0.99 ± 0.21	7.21E-09	0.87 ± 0.13	1.08 ± 0.30	4.83E-08
eGFR (ml/min), mean ± SD	/	/	/	101.01 ± 14.33	105.30 ± 20.66	5.17E-02
Enzymatic CRE (μmol/L), mean ± SD	8394.00 ± 5658.00	9760.00 ± 7047.00	5.70E-02	/	/	/
BUN (mmol/L), mean ± SD	/	/	/	5.61 ± 1.17	6.64 ± 2.41	7.17E-01
UA (mmol/L), mean ± SD	/	/	/	0.32 ± 0.09	0.27 ± 0.07	6.85E-01

^1^Self-reported MG.

MG, myasthenia gravis; HC, health control; AChR, acetylcholine receptor; Cys-C, Cystatin C; eGFR, estimated glomerular filtration rate; BUN, blood urea nitrogen; UA, uric acid; CRE, creatinine.

Given the potential confounding effects from the immunosuppressive therapies, 35 treatment-naive CHN gMG patients were subsequently selected, with sex and age comparable to those of HCs (Sex, p = 9.80E-01; Age, p =3.55E-01) (**Table 3**). Treatment-naive MG patients exhibited significantly elevated serum Cys-C levels compared to HCs (naive CHN gMG vs. HCs, 1.04 ± 0.26 vs. 0.87 ± 0.13mg/L, p = 8.19E-03), while other renal function-related indicators were remained comparable, including eGFR (naive CHN gMG vs. HCs, 102.50 ± 12.66 vs. 100.40 ± 12.46ml/min, p = 5.93E-01), UA (naive CHN gMG vs. HCs, 0.30 ± 0.12 vs. 0.32 ± 0.09mmol/L, p = 5.36E-01), BUN (naive CHN gMG vs. HCs, 6.50 ± 2.21 vs. 5.61 ± 1.17mmol/L, p = 1.33E-01), further confirming that the elevation of serum Cys-C in MG patients was unrelated to renal function impairment (**Figure 2B**).

**Table 3:** Clinical characteristics and renal function-related indicators in treatment-naive MG patients.

Feature	HC (n=70)	Naive MG (n=35)	p value
Sex, % men/% women	43.15%/56.85%	42.86%/57.14%	9.80E-01
Age (years), mean ± SD	48.13 ± 13.40	51.90 ± 15.51	3.55E-01
AChR positive, n (%)	/	30 (100%)	/
Cys-C (mg/L), mean ± SD	0.87 ± 0.13	1.04 ± 0.26	8.19E-03
eGFR (ml/min), mean ± SD	100.40 ± 12.46	102.50 ± 12.66	5.93E-01
Enzymatic CRE (μmol/L), mean ± SD	5.61 ± 1.17	6.50 ± 2.21	1.33E-01
BUN (mmol/L), mean ± SD	0.32 ± 0.09	0.30 ± 0.12	5.36E-01

MG, myasthenia gravis; HC, health control; AChR, acetylcholine receptor; Cys-C, Cystatin C; eGFR, estimated glomerular filtration rate; BUN, blood urea nitrogen; UA, uric acid.

### 3.3 Serum Cys-C correlates with disease severity in patients with MG

**Figure 3:**
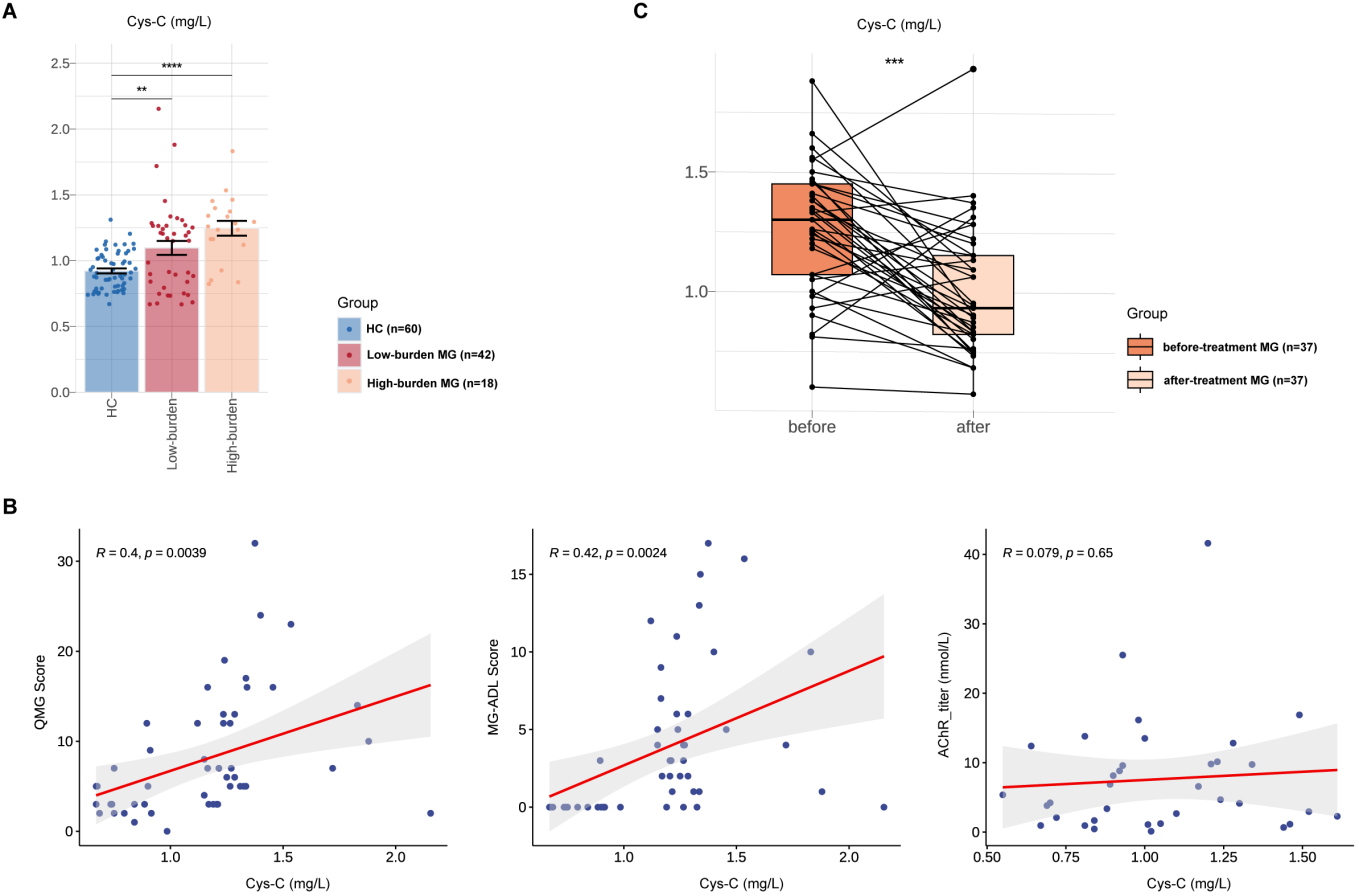
Associations between Serum Cys-C and MG disease severity in Chinese MG cohorts. Comparison of serum Cys-C levels between low-burden MG (N=42), high-burden MG (N=42) and HCs (N=60). **(B)** Correlations between serum Cys-C levels and indicators associated with MG, including QMG score, MG-ADL score and AChR titers. **(C)** Comparative analysis of serum Cys-C level in a subset of MG patients (N = 37) before and after treatment. MG, myasthenia gravis; QMG, quantitative myasthenia gravis score; MG-ADL, MG-activities of daily living profile; AChR, acetylcholine receptor; Cys-C, Cystatin C. “**” indicates p < 0.01, “***” indicates p < 0.001, and “****” indicates p < 0.0001.

Based on MG-ADL scores, MG patients were stratified into high-disease burden (MG-ADL score ≥6, N = 18) and low-disease burden (MG-ADL score <6, N=42) groups at enrollment (**Table 4**) (19). Serum Cys-C levels were higher in the high-burden group than in the low-burden group (High burden MG vs. HCs, 1.12 ± 0.29 vs. 1.04 ± 0.27, p =6.10E-02), and both groups had significantly elevated Cys-C levels compared to HCs (High burden MG vs. HCs, 1.12 ± 0.29 vs. 0.92 ± 0.14, p = 4.47E-05; Low burden MG vs. HCs, 1.04 ± 0.27 vs. 0.92 ± 0.14, p = 6.00E-03) (**Figure 3A**).

**Table 4:** Clinical characteristics MG patients with low and high disease burden.

Basic characteristic	HC (n=60)	Low-burden MG (n=42)	High-burden MG (n=18)	p value
Sex, % men/% women	41.67%/58.33%	42.86%/57.14%	50.00%/50.00%	9.71E-01
Age (years), mean ± SD	42.10 ± 16.54	42.80 ± 16.45	43.67 ± 11.81	5.57E-01
AChR positive, n (%)	/	42 (100.00%)	18 (100.00%)	/
Thymoma, n (%)	/	10 (23.81%)	7 (28.00%)	3.48E-01
Thymectomy, n (%)	/	6 (14.29%)	2 (8.00%)	9.99E-01
Generalized MG, n (%)	/	42 (100.00%)	18 (100.00%)	/
EOMG, n (%)	/	24 (57.14%)	8 (44.44%)	4.09E-01
LOMG, n (%)	/	8 (19.05%)	3 (16.67%)	7.24E-01
TAMG, n (%)	/	10 (23.81%)	7 (38.89%)	3.49E-01
QMG, mean ± SD	/	5.64 ± 4.20	19.28 ± 7.66	<0.001
MG-ADL, mean ± SD	/	1.60 ± 1.98	14.17 ± 5.33	<0.001
MG-QOL15r, mean ± SD	/	5.14 ± 7.48	27.27 ± 15.55	<0.001
MGC, mean ± SD	/	2.93 ± 3.78	26.78 ± 12.58	<0.001

MG, myasthenia gravis; HC, health control; AChR, acetylcholine receptor; EOMG, early-onset myasthenia gravis with AChR antibody; LOMG, late-onset myasthenia gravis with AChR-Ab; TAMG, thymoma-associated myasthenia gravis; QMG, quantitative myasthenia gravis score; MG-ADL, MG-activities of daily living profile; MG-QOL15r, myasthenia gravis quality of life 15-item revised; MGC, myasthenia gravis composite.

We then analyzed the associations between serum Cys-C and clinical severity of MG, as measured by the MG-QMG and MG-ADL scores. Serum Cys-C levels correlated significantly with advanced MG-QMG scores (R=0.40, p = 3.90E-03) and MG-ADL scores (R=0.42, p = 2.40E-03. No correlation was observed between serum Cys-C levels and AChR antibody titers (R=0.08, p = 6.50E-01) (**Figure 3B**). A comparative analysis of the same cohort of 37 patients pre- and post-treatment revealed a significant reduction in serum Cys-C levels following treatment (1.26 ± 0.27 to 1.00 ± 0.27; p < 0.001) (**Figure 3C**). Among the cohort of 37 patients, all MG patients received pyridostigmine and steroids. Additionally, some patients underwent treatment with oral immunosuppressants (tacrolimus, mycophenolate mofetil, and azathioprine), targeted therapies (efgartigimod, eculizumab, and rituximab), as well as rescue therapies (intravenous immunoglobulin and plasma exchange) (**Supplementary Table 2**).

### 3.4 Serum Cys-C levels are associated with the Th2 conversion to Th1 in MG

**Figure 4:**
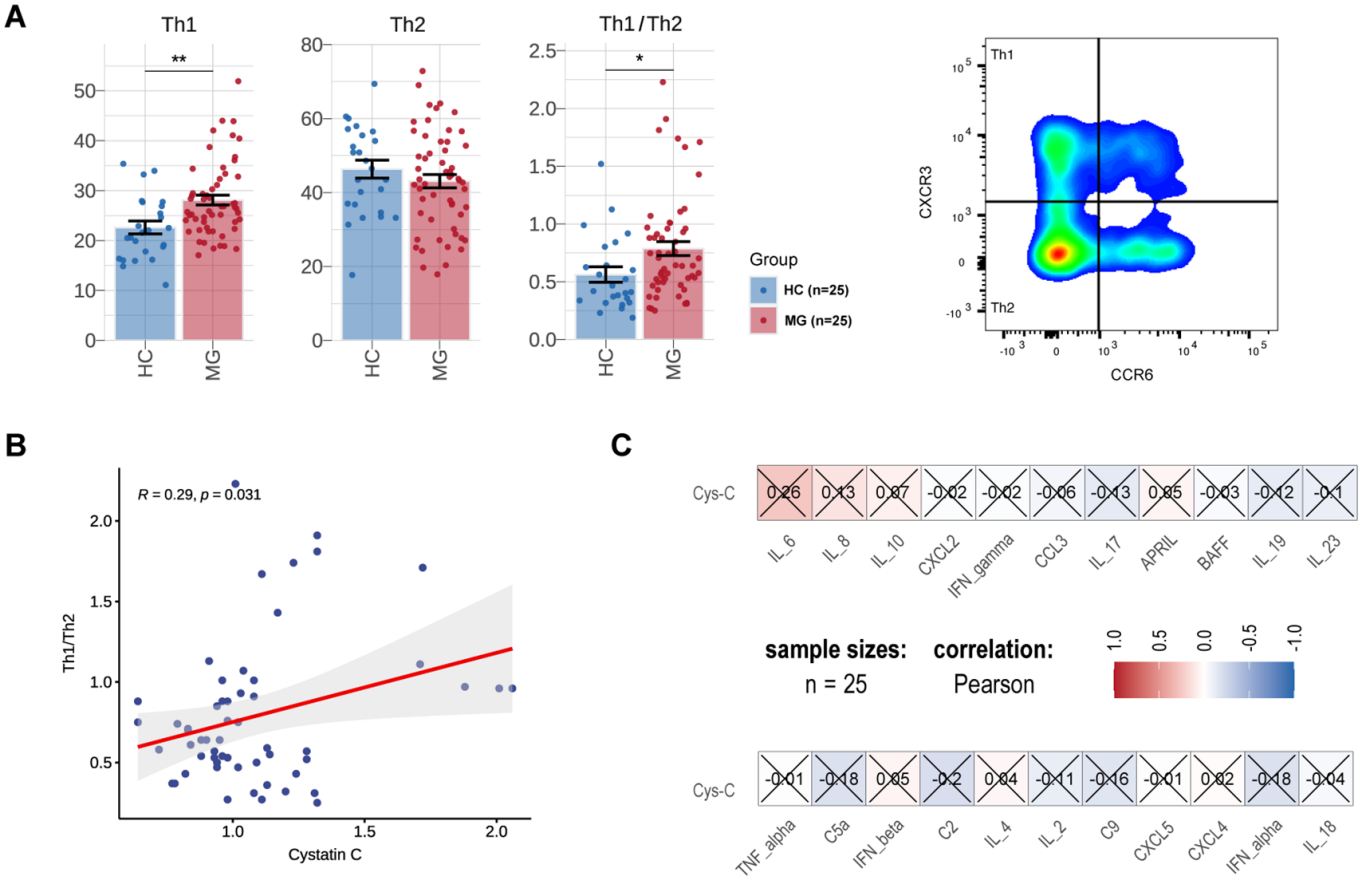
Associations between serum Cys-C and inflammatory markers in Chinese MG cohorts. Comparison of the proportion of Th1 cells and Th2 cells of T lymphocytes, Th1/Th2 ratio between MG (N=25) and HCs (N=25). **(B)** Correlations between Th1/Th2 ratio and serum Cys-C levels in MG. **(C)** Correlations between cytokines/chemokines and serum Cys-C levels in MG. MG, myasthenia gravis; HC, health control; Th, T helper cell; Cys-C, Cystatin C. “*” indicates p < 0.05, “**” indicates p < 0.01.

Cys-C has been implicated in switching the inefficient Th2 cytokine response to an effective Th1 response, potentially involved in the regulation of autoimmunity (25). To further explore its role in MG pathogenesis, we analyzed the proportion or count of T lymphocyte subsets and MG-related cytokines in a subgroup of 25 gMG patients. Compared to HCs, MG patients exhibited a significantly increased proportion of Th1 cells (MG vs. HCs, 28.12 ± 7.45 vs. 22.63 ± 6.27, p = 1.37E-03), while the proportion of Th2 cells remained unchanged (MG vs. HCs, 43.07 ± 13.39 vs. 46.34 ± 11.95, p = 2.85E-01) (**Figure 4A**). In addition, the Th1/Th2 ratio was markedly elevated in gMG patients (MG vs. HCs, 0.79 ± 0.45 vs. 0.56 ± 0.33, p = 1.48E-02) and showed a significant positive correlation with serum Cys-C levels (R=0.29, p = 3.10E-02) (**Figure 4B**). Furthermore, the differentiation of functional effectors such as Th1 and Th2 cells from T cells are subject to the regulation of cytokines (26). Compared with HCs, MG patients exhibited an elevation in plasma inflammatory molecules, including IL-6, IL-8 and IL-10, though differences did not reach statistical significance (**Figure 4C**).

## 4 Discussion

In this study, we analyzed MG-related data from the UKB cohort for the first time and investigated Cys-C as a potential biomarker candidate for MG. Our findings revealed significantly elevated serum Cys-C levels in MG patients, a result consistently validated across different cohorts and populations. Furthermore, our analysis demonstrated a significant correlation between Cys-C levels and MG disease severity. Notably, Cys-C levels were associated with the shift from Th2 to Th1 immune responses, a process known to contribute to the pro-inflammatory environment in MG, thereby providing insights into its role in MG pathogenesis (27).

Cystatins, constituting a large group of evolutionarily conserved proteins, function mainly as reversible, tight-binding inhibitors of cysteine proteases (cathepsins), and are involved in several immune processes controlling the cathepsins (28). Cathepsins play crucial roles in oncogenesis and autoimmune diseases, primarily through activation of inflammatory responses and promotion of extracellular matrix (ECM) degradation (29). Cys-C is a classical and potent cysteine protease inhibitor that belongs to the family II of the cystatin super-family, and is produced and released at a constant rate by all nucleated cells. It is critical for controlling the cleavage and removal of the MHC class II invariant chain (30, 31).

Clinically, Cys-C is widely used as a biomarker of renal functions due to its relatively low molecular weight and ease of detection (25). In systemic lupus erythematosus (SLE), Cys-C is a promising marker for monitoring organ damage and disease activity in SLE (32). Growing evidence suggests the direct involvement of Cys-C in various immunologic disorders, with its encoding gene regulating cytokine expression under inflammatory or infectious conditions (33, 34). Previous studies have shown that Cys-C downregulation enhances human leukocyte antigen (HLA) class II expression in macrophages, increases CD4+ T lymphocyte proliferation, and enhances IFN-γ secretion (35). Conversely, another study concluded that elevated Cys-C levels in IFN-γ induced macrophages shift the immune responses toward protective Th1 immunity (25). Meanwhile, Hashimoto et al. observed significant downregulation of Cys-C transcripts upon dendritic cells maturation, a process essential for T cell activation and the bridge between innate and adaptive immunity during acute infection (25, 36). Cys-C exhibits context-dependent roles in autoimmunity. While it suppresses cathepsins to limit inflammation, elevated Cys-C in MG paradoxically correlates with immune dysregulation. This may stem from its dual immunomodulatory effects: promoting Th1 responses in macrophages (25) yet exacerbating Th1/Th17-driven pathology in MG (37). Our data linking higher Cys-C to increased Th1/Th2 ratios suggest it amplifies autoimmune pathways via Th2-to-Th1 conversion. Future studies should target tissue-specific protease-immunity crosstalk to restore homeostasis in MG.

Collectively, these findings indicate a critical role for Cys-C in the immunoregulation of T cell subsets. A previous study using an experimental autoimmune MG (EAMG) model demonstrated that increased proportions of Th1 and Th17 cells exacerbate MG pathogenesis (37). Given the pivotal role of T lymphocytes in MG pathogenesis, we hypothesize that Cys-C regulates the Th2-to-Th1 conversion via various cytokines, thereby influencing the pathogenesis of MG. In our study, a significant positive correlation was observed between Cys-C levels and the Th1/Th2 ratio, suggesting that elevated Cys-C levels promote the conversion of an inefficient Th2 cytokine response to an effective Th1 response in MG.

Beyond its role in Th2-to-Th1 conversion, Cys-C may contribute to the pathogenesis of MG through additional mechanisms. One potential pathway involves the modulation of antigen-presenting cell function by disrupting the activity of cysteine proteases, which can lead to abnormal self-antigen presentation and the activation of autoreactive T cells (38). Another mechanism is its ability to amplify complement-mediated synaptic damage by regulating the complement cascade in a protease-dependent manner, particularly through its interaction with complement component C4 (39). This evidence highlights the intricate roles of Cys-C in immune regulation and tissue damage in MG, underscoring the importance of further research into therapeutic approaches that target protease-mediated immune system interactions.

Currently, few MG biomarkers have been identified as correlated with disease severity. While flow cytometry-based markers representing autoreactive T-cell activation are valuable, they require substantial time and resources for evaluation. Serum Cys-C serves as a more efficient and robust biomarker for monitoring disease progression, thereby making it a promising candidate for widespread clinical application.

This study has several limitations that warrant consideration: First, elevated Cys-C expression is associated with inflammatory autoimmune diseases and tumor development (25), which may limit its specificity as a biomarker for MG. Second, the underlying molecular mechanisms by which Cys-C contributes to MG pathogenesis were not investigated, leaving its precise role in disease progression unclear. Moreover, the retrospective design of this study introduces potential selection bias, which may limit the generalizability of our findings to broader populations. Finally, the small sample size reduces the statistical power and increases the risk of bias.

## 5 Conclusion

Serum Cys-C levels were significantly elevated in AChR-Ab positive gMG patients and positively correlated with disease severity and the Th1/Th2 ratio. These findings suggest that serum Cys-C may serve as a potential biomarker for monitoring disease progression for MG.

## Data Availability

The original contributions presented in the study are included in the article/**Supplementary Material**. Further inquiries can be directed to the corresponding authors.

## Supplementary material

Supplementary Table 1 Screening results of MG metabolic biomarkers in UKB.

Supplementary Table 2 Treatment options for 37 MG patients.
